# The Effect of Alternating Current Iontophoresis on Rats with the Chronic Constriction Injury to the Infraorbital Nerve

**DOI:** 10.1155/2012/405292

**Published:** 2012-05-23

**Authors:** Yoko Yamazaki, Masahiro Umino, Haruhisa Fukayama, Masahiko Shimada

**Affiliations:** ^1^Department of Orofacial Pain Management, Tokyo Medical and Dental University, Yushima, Bunkyo-ku, Tokyo 113-8549, Japan; ^2^TTI ellebeau, Inc., Shinkan Building, Higashi-shinagawa, Shinagawa-ku, Tokyo 140-0002, Japan; ^3^Department of Anesthesiology and Clinical Physiology, Tokyo Medical and Dental University, Yushima, Bunkyo-ku, Tokyo 113-8549, Japan

## Abstract

This study aimed to examine the effect of AC iontophoresis on rats with the chronic constriction injury (CCI) to the infraorbital nerve by animal experiments. CCI model rats were divided into four groups, namely, rats that received general anesthesia for 60 min except AC IOP (CCI: *n* = 5), AC IOP with 0.9% physiological saline for 60 min (CCI + saline AC IOP: *n* = 5), AC IOP with 4% lidocaine hydrochloride for 60 min (CCI + lidocaine AC IOP: *n* = 5), and attachment of two electrodes soaked with 4% lidocaine hydrochloride to the facial skin for 60 min (CCI + attach lidocaine: *n* = 5). In the CCI + lidocaine AC IOP group, an elevated withdrawal threshold was observed after AC IOP, and the duration of efficacy was longer compared with that in the CCI + saline AC IOP and CCI + attached lidocaine groups. A significant decrease in the number of Fos-like immunoreactive (LI) cells was observed in the CCI + lidocaine AC IOP group compared with that in the CCI group. These findings suggest that the effect of CCI + lidocaine AC IOP group may be caused by active permeation of lidocaine into the facial skin and electrical stimulation of the trigeminal nucleus.

## 1. Introduction

**Figure 1:**
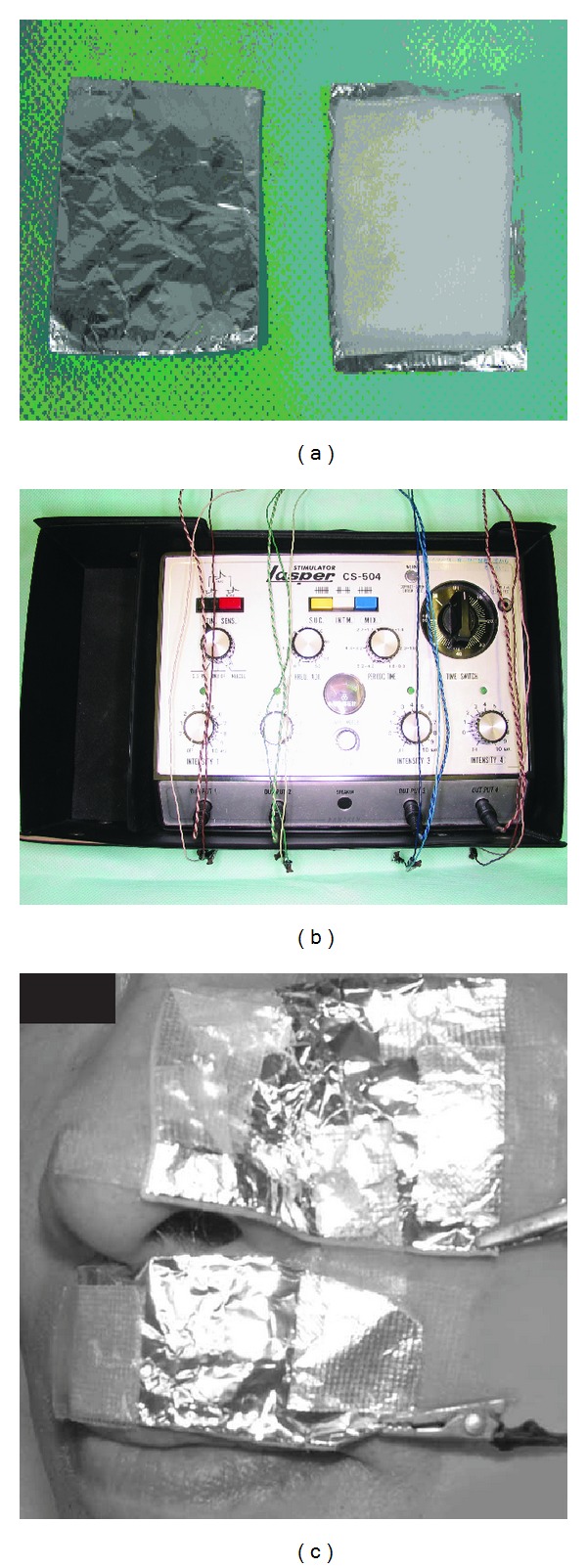
The electrodes were made of cotton and aluminum foil (a). A pulsed alternating current (AC) voltage generator was used (LASPER; Sankyo Electric Co., Japan) (b). A photograph of application to a patient (c).

It is difficult to control pain caused by nerve injury with medications such as anti-inflammatory agents and antibiotics. Medications recommended for neuropathic pain are antidepressants, calcium channel a2-d ligands, opioid agonists, tramadol, and so on [1]. In our clinic, many medicines such as antidepressants, anticonvulsants, and antianxiety medications are usually used together with cognitive-behavioral treatment to treat these patients. *Kampo *(Japanese traditional medicine) and acupuncture therapy are also used. In addition, we provide photodynamic therapy and alternating current (AC) iontophoresis (Figure 1).

Transdermal drug delivery can be enhanced by chemical and physical enhancement systems. Iontophoresis is one of the physical enhancement systems using electrical energy [2, 3]. Two electrodes are placed apart from each other on the skin, and charged drugs are transported through the skin by electrophoresis. Electroosmosis generated within the skin by electric current also enables transport of hydrated water ions inside the skin [4, 5].

The principle of iontophoresis was first described by Veratti in 1747, and in the last century, experiments were performed using a range of different pharmaceutical agents. In 1975, Echols et al. investigated the effect of iontophoresis on the mucosa of the inner ears of guinea pigs using 4% lidocaine hydrochloride [6], and researches in the fields of otolaryngology and dentistry mainly focused on its application as a method of local anesthesia.

Direct current (DC) is normally used in iontophoresis; however, it often causes skin irritations, burns, and inflammation [7]. In addition to its side effects, the fact that electrode polarization often occurs during DC iontophoresis suggests that the method cannot be applied for longer durations [8, 9]. To overcome these side effects, Reinauer et al. suggested the use of AC iontophoresis in 1993 [10]. AC iontophoresis causes little skin irritation and does not result in electrode polarization; therefore, it can be used for longer duration than DC iontophoresis. Taking advantage of these characteristics, we clinically apply AC iontophoresis with 4% lidocaine hydrochloride in our department to treat neuropathic pain.

 With respect to the mechanism of enhancing percutaneous absorption by AC iontophoresis, Shibaji et al. hypothesized in 2001 that the decrease of effective Stokes radius of the ion with an increase in the vibration energy of the ion caused by alternating electric potential applied between electrodes plays a decisive role in ion transportation [11].

 Although AC iontophoresis has been confirmed to be clinically effective, the mechanism of action is still unclear. In the present study, the effect of AC iontophoresis was examined in rat experiments. 

## 2. Materials and Methods

 Animal experiments were conducted using chronic constriction injury (CCI) as a model of neuropathic pain to evaluate the effect of AC iontophoresis.

### 2.1. Animals

Forty-five male Sprague-Dawley rats (240 g~300 g) were used. The rats were housed under a 12-hour light/dark cycle with food and water available *ad libitum*. The rats were treated according to the guidelines of the International Association for the Study of Pain [12]. This study was approved by the Institutional Animal Care and Use Committee of Tokyo Medical and Dental University (no. 0080093).

### 2.2. Chronic Constriction Injury (CCI) Model Rats

Withdrawal threshold was measured by using von Frey monofilaments before the operation. CCI was performed using the method of Imamura et al. [13]. The rats were anesthetized with pentobarbital sodium [50 mg/kg intraperitoneally (i.p.)]; an incision was made intraorally, and the left infraorbital nerve was exposed. Two loosely constrictive ligatures were tied around the nerve with 4.0 chromic gut sutures. Following this, the incision was sutured using 4.0 silk.

The withdrawal threshold was again measured 7 days after the operation and compared with data before the operation. The that rats showed significant decrease withdrawal threshold were recognized as the CCI model rats.

The sham operation was the same as the CCI operation except for the nerve ligature (*n* = 5).

### 2.3. AC Iontophoresis (AC IOP)

The electrical stimulator used for AC IOP was a clinical device (LASPER^*©*^ CS-504; Sankyo Electric Co., Japan). The electrode was made of cotton and a copper wire mesh. One electrode was soaked by 0.05 mL of 4% lidocaine hydrochloride or 0.9% physiological saline. Two electrodes were placed on the facial skin of the maxillary nerve area and fixed with surgical tape, and a pulsed AC voltage (1 V, 50 Hz) was applied for 60 min. 

### 2.4. Behavioral Experiment

The CCI model rats were divided into four groups, namely, rats that received anesthesia for 60 min except AC IOP (CCI group, *n* = 5), AC IOP with 0.9% physiological saline applied to the rats for 60 min (CCI + saline AC IOP group, *n* = 5), AC IOP with 4% lidocaine hydrochloride applied to the rats for 60 min (CCI + lidocaine AC IOP group, *n* = 5), and placement of two electrodes soaked with 4% lidocaine hydrochloride on the facial skin for 60 min (CCI + attach lidocaine group: *n* = 5).

The mechanical withdrawal threshold was measured prior to anesthesia using von Frey monofilaments. Mechanical stimulation was applied to the whisker pad and alternately applied to the contralateral (right) and ipsilateral (left) sides. The mechanical withdrawal threshold was determined as the lowest force that caused a head withdrawal response. Each monofilament was applied three times at intervals of 5 sec. If a rat showed a head withdrawal response or grooming of the whisker pads, even one of the three times, the corresponding force was considered to cause a withdrawal response. Mechanical withdrawal thresholds were measured five times, and the median of the five values was determined to be the mechanical withdrawal threshold of the rat. These values were then averaged for each group. The mechanical withdrawal threshold in the CCI group was compared with that in the sham group to demonstrate that the change in the withdrawal threshold was not caused by inflammation because of the operation.

The rats were anesthetized with pentobarbital sodium (50 mg/kg i.p.), and two electrodes were placed on the facial skin of the maxillary nerve area and fixed with surgical tape. General anesthesia was maintained with 1%~4% halothane. Each group was treated for 60 min. The mechanical withdrawal threshold was measured at 0, 30, 60, 90, and 120 min after treatment. 

### 2.5. Immunohistological Experiment

Twenty CCI model rats were divided into four groups as seen for the behavioral experiments. After treatment, the mechanical stimulation (60 g, 0.5 Hz, 10 min) was applied to the rats with a von Frey monofilament. Two hours later, the rats were anesthetized with pentobarbital sodium (80 mg/kg i.p.) and perfused through the aorta with 0.9% physiological saline 300 mL and 4% paraformaldehyde in 0.1 M phosphate buffer (PB) 300 mL. The brain and medulla were removed, and immersed in 4% paraformaldehyde in 0.1 M PB for 3 days at 4°C, and transferred to 30% sucrose (w/v) in 0.1 M PB for several days for cryoprotection.

The sections were cut on a freezing microtome in thickness of 30 *μ*m, and every fourth section was collected in 0.02 M phosphate buffer saline (PBS). Free-floating sections were washed in PBS and then treated with 3% hydrogen peroxide (H_2_O_2_) in PBS for 10 min. After three washes with PBS, the sections were incubated with 3% normal goat serum (NGS) for 2 hours. Following this, the sections were incubated with rabbit anti-Fos (1 : 10,000; c-fos ab-5, Oncogene, Cambridge, MA, USA) in 3% NGS overnight at 25°C. After three washes with PBS, the sections were incubated with biotinylated secondary IgG (1 : 200; Vector Labs, Burlingame, CA, USA) for 2 hours at 25°C. After three washes with PBS, the sections were incubated with peroxidase-conjugated avidin-biotin complex (1 : 100; ABC, Vector Labs) for 2 hours at 25°C. Following three rinses with PBS and one rinse with 0.05 M Tris-buffer (TB), the sections were immersed in 0.035% 3,3′-diaminobenzidine-tetra HCl (DAB, Sigma, St. Louis, MO, USA), 0.2% nickel ammonium sulfate, and 0.05% peroxide in 0.05 M TB. Finally, the sections were rinsed in PBS, mounted on gelatin-coated slides dehydrated in alcohol, cleared in xylene, and covered with Eukitt (O. Kindler, Freiburg, Germany).

The cells with black deposits in the nuclei were considered to be Fos-like immunoreactive (LI) cells. The number of Fos-LI cells was counted per section per rat. The mean number of Fos -LI cells was calculated for five rats.

### 2.6. Analyses

 Data are presented as means ± standard error of the mean (SEM). One-way ANOVA was used to analyze the difference between the groups in the behavioral and immunohistological experiments. Post hoc comparisons were performed with Bonferroni test to assess the mechanical withdrawal thresholds at 7 days after CCI. And post hoc comparisons were performed with Dunnett test to assess the change in the mechanical withdrawal thresholds after treatments and distribution of expression of Fos-LI cells in the trigeminal nucleus. *P* values of less than 0.05 were considered statistically significant.

## 3. Result

**Figure 2:**
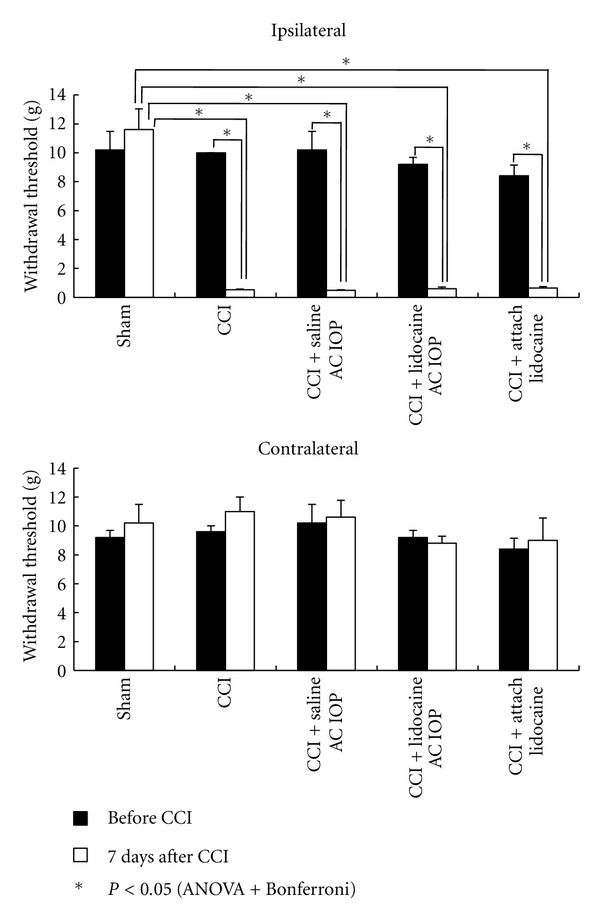
Change in mechanical withdrawal thresholds 7 days after chronic constriction injury (CCI) (mean ± SEM). The values of 7 days after CCI decreased significantly on the ipsilateral side compared with the preoperative values. In addition, a significant difference was observed between the sham group and other groups. *P < 0.05, *n* = 5.

**Figure 3:**
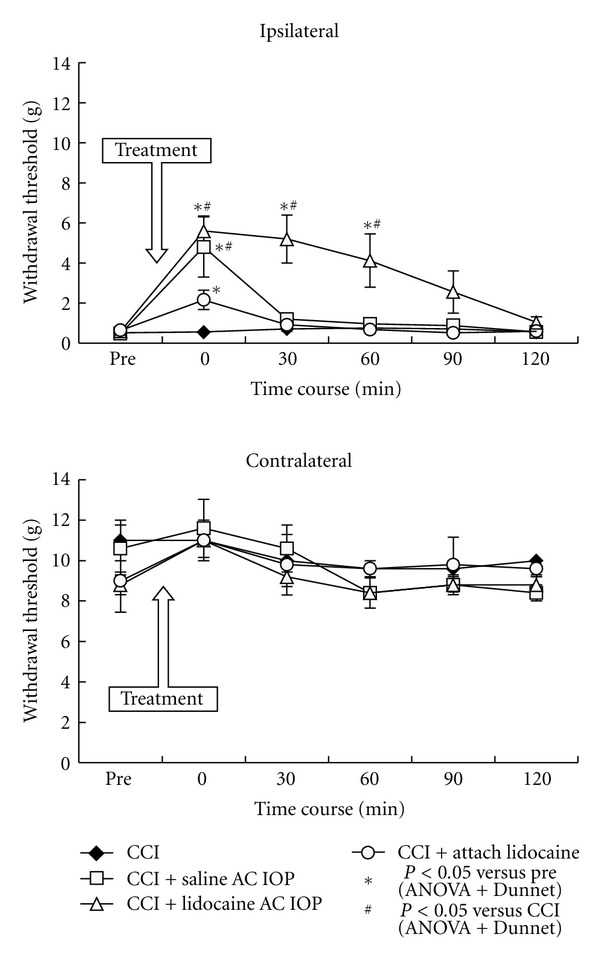
After 60 min treatment, mechanical withdrawal thresholds were measured at 0, 30, 60, 90, and 120 min. A significant increase in the withdrawal threshold was observed in the CCI + lidocaine AC IOP group until 60 min after treatment. The value in the CCI + saline AC IOP group at 0 min also increased significantly. The values in the CCI + attach lidocaine group at 0 min increased significantly compared with those prior to AC IOP; however no significant difference was observed compared with the values in the CCI group. All the changes were observed on the ipsilateral side. ^∗,#^ *P* < 0.05, *n* = 5.

 Mechanical withdrawal thresholds in all the groups of rats that received CCI decreased significantly on the ipsilateral side compared with those in the sham group (Figure 2). In the CCI + lidocaine AC IOP group, the withdrawal threshold during the behavioral experiments was elevated after AC IOP, and the duration of efficacy was longer compared with that in the CCI + saline AC IOP group and the CCI + attach lidocaine group (Figure 3).

**Figure 4:**
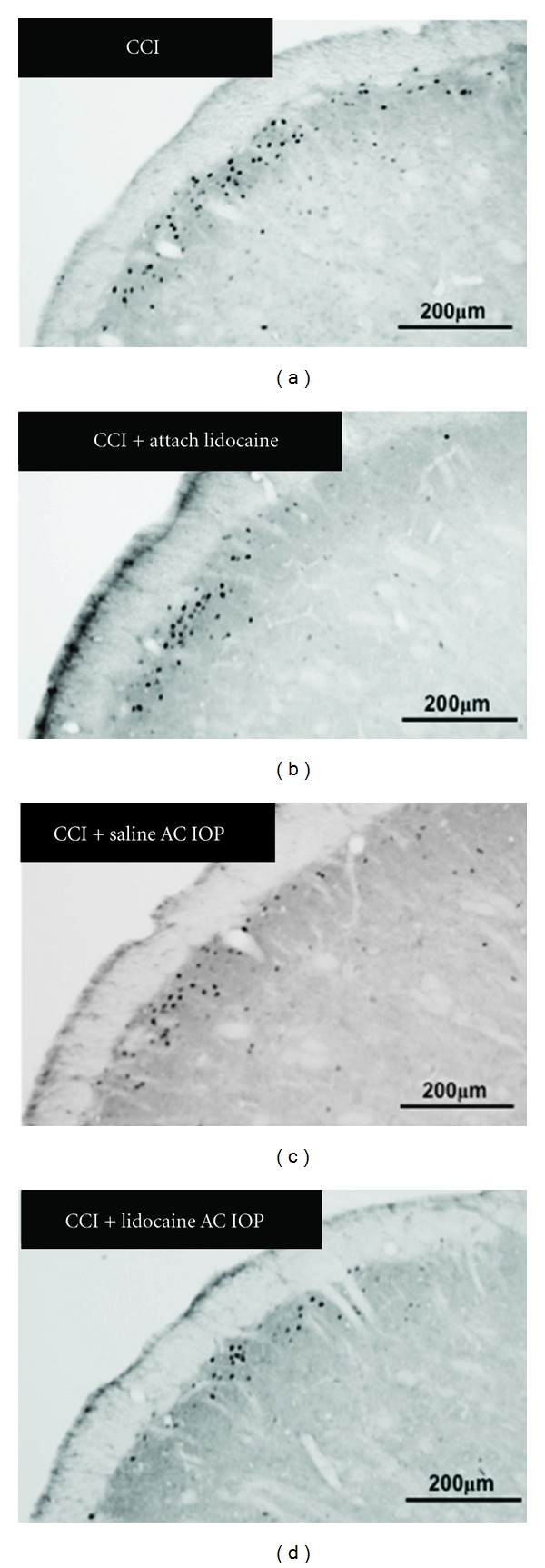
Expression of Fos-LI cells in the trigeminal subnucleus caudalis. A small number of Fos-LI cells was observed in the CCI + lidocaine AC IOP group.

**Figure 5:**
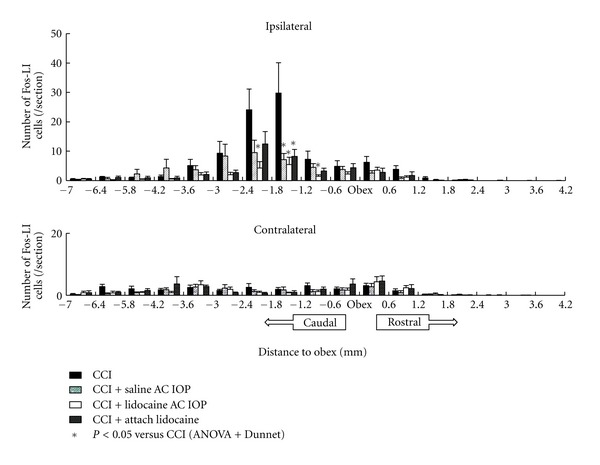
Distribution of the expression of Fos-LI cells in the trigeminal nucleus. The CCI + lidocaine AC IOP group showed a decrease in the number of Fos-LI cells from −2.4 mm to −0.6 mm in the trigeminal nucleus. The number of Fos-LI cells decreases from −1.8 mm to −1.2 mm in the trigeminal nucleus of the CCI + saline AC IOP group and the CCI + attach lidocaine group. No significant difference in the number of Fos-LI cells was observed on the contralateral side. *P < 0.05, *n* = 5.

 The immunohistological experiment revealed expression of Fos-LI cells in the trigeminal spinal nucleus (Figures 4 and 5). In the CCI group, the expression of numerous Fos-LI cells was observed in an area 1.2 mm cranial and 3.6 mm caudal from the obex. In the CCI + lidocaine AC IOP group, a significant decrease was observed in the expression of Fos-LI cells compared with that in the CCI group in an area 0.6 mm caudal to 2.4 mm caudal from the obex. The location of this zone corresponds to the trigeminal subnucleus caudalis. A decrease in the number of Fos-LI cells was also observed in an area 1.2 mm caudal to 1.8 mm caudal from the obex in the CCI + saline AC IOP and CCI + attach lidocaine group.

 Thus, the expression of Fos-LI cells was suppressed over a wide area in the CCI + lidocaine AC IOP group compared with that in the other two groups.

## 4. Discussion

 Nerve fibers enter inside the teeth through the jawbone, and many dental procedures can potentially affect these nerve fibers. Indeed, dental treatment may occasionally result in neuropathic pain [14, 15].

Neuropathic pain has been treated by various methods. In our department, we usually employ AC iontophoresis as one of the methods to manage neuropathic pain [16–19].

Iontophoresis is a method of utilizing electrical energy to transport drugs through the skin. DC iontophoresis has been widely used in various fields such as ophthalmic surgery, anesthesia of the tympanic membrane, diagnosis of cystic fibrosis, and treatment of hyperhidrosis [20–23]. Although commercial devices are available, DC iontophoresis creates some problems such as skin burns [7] and electrode polarization [8, 9].

Therefore, the AC iontophoresis is used to treat orofacial pain in our clinic to avoid these disadvantages of DC iontophoresis.

In the present study, an elevated withdrawal threshold and a reduced number of Fos-LI cells were observed in the CCI + saline AC IOP group and CCI + lidocaine AC IOP group. The saline did not have a local anesthesia effect, indicating that electrical stimulation may have exerted some effect on the central pain pathway.

The duration of elevation of the withdrawal threshold in the CCI + lidocaine AC IOP group was longer compared with that in the CCI + saline AC IOP group and CCI + attach lidocaine group. The effect of 4% lidocaine hydrochloride AC iontophoresis treatment lasted over several days in our clinical case [12, 13]. The effect of CCI + saline AC IOP group may have been caused by electrical stimulation of the trigeminal nucleus, and the effect of CCI + attach lidocaine group may have been caused by passive diffusion of the lidocaine into the facial skin. Kinoshita et al. reported that the transdermal delivery of lidocaine by AC iontophoresis has a possibility to use for local anesthesia and the pain management of the skin [24]. Therefore, AC iontophoresis probably allows lidocaine to pass actively through the skin. These findings suggest that the effect of CCI + lidocaine AC IOP group may have been caused by active diffusion of lidocaine through the facial skin and electrical stimulation of the trigeminal nucleus.

Thus, this study suggests that AC iontophoresis has a possibility to be a useful treatment for patients suffering from neuropathic pain caused by oral and maxillofacial surgery.

Recently, many studies have been conducted on the AC iontophoresis. These studies demonstrated that lidocaine transport depends on voltage, time, and frequency [25, 26]. Studies of electrical waveforms and electrodes have also been conducted [27, 28]. However, further studies are needed to provide safe and effective AC iontophoresis therapy.

## References

[1] Dworkin RH, O’Connor AB, Audette J (2010). Recommendations for the pharmacological management of neuropathic pain: an overview and literature update. *Mayo Clinic Proceedings*.

[2] Higo N (2007). The recent trend of transdermal drug delivery system development. *Yakugaku Zasshi*.

[3] Kalia YN, Naik A, Garrison J, Guy RH (2004). Iontophoretic drug delivery. *Advanced Drug Delivery Reviews*.

[4] Guy RH, Kalia YN, Delgado-Charro MB, Merino V, López A, Marro D (2000). Iontophoresis: electrorepulsion and electroosmosis. *Journal of Controlled Release*.

[5] Pikal MJ (2001). The role of electroosmotic flow in transdermal iontophoresis. *Advanced Drug Delivery Reviews*.

[6] Echols DF, Norris CH, Tabb HG (1975). Anesthesia of the ear by iontophoresis of lidocaine. *Archives of Otolaryngology*.

[7] Ledger PW (1992). Skin biological issues in electrically enhanced transdermal delivery. *Advanced Drug Delivery Reviews*.

[8] Chien YW, Siddiqui O, Sun Y, Shi WM, Liu JC (1987). Transdermal iontophoretic delivery of therapeutic peptides/proteins. I: insulin. *Annals of the New York Academy of Sciences*.

[9] Chien YW, Lelawongs P, Siddiqui O, Sun Y, Shi WM (1990). Facilitated transdermal delivery of therapeutic peptides and proteins by iontophoretic delivery devices. *Journal of Controlled Release*.

[10] Reinauer S, Neusser A, Schauf G, Holzle E (1993). Iontophoresis with alternating current and direct current offset (AC/DC iontophoresis): a new approach for the treatment of hyperhidrosis. *British Journal of Dermatology*.

[11] Shibaji T, Yasuhara Y, Oda N, Umino M (2001). A mechanism of the high frequency AC iontophoresis. *Journal of Controlled Release*.

[12] Zimmermann M (1983). Ethical guidelines for investigations of experimental pain in conscious animals. *Pain*.

[13] Imamura Y, Kawamoto H, Nakanishi O (1997). Characterization of heat-hyperalgesia in an experimental trigeminal neuropathy in rats. *Experimental Brain Research*.

[14] Tay ABG, Zuniga JR (2007). Clinical characteristics of trigeminal nerve injury referrals to a university centre. *International Journal of Oral and Maxillofacial Surgery*.

[15] Pogrel MA, Thamby S (1999). The etiology of altered sensation in the inferior alveolar, lingual, and mental nerves as a result of dental treatment. *Journal of the California Dental Association*.

[16] Shibaji T, Kawashima M, Mashu S (2006). AC iontophoresis for orofacial pain and abnormal sensations. *Anesthesia Progress*.

[17] Shibaji T, Umino M, Shimada M AC iontophoresis for neuropathic pain in the tongue caused by oral urgery.

[18] Shibaji T, Umino M, Shimada M AC iontophoresis for neuropathic pain caused by orofacial surgery.

[19] Yamazaki Y, Shibaji T, Takahashi T (2007). The effect of 4%lidocine AC iontophoresis for postherpetic neuralgia. *The Journal of the Japanese Society for the Study of Chronic Pain*.

[20] Meyer DR, Linberg JV, Vasquez RJ (1990). Iontophoresis for eyelid anesthesia. *Ophthalmic Surgery*.

[21] Nagai T, Nagai M, Nagata Y, Morimitsu T (1989). The effects of anesthesia of the tympanic membrane on eustachian tube function. *Archives of Oto-Rhino-Laryngology*.

[22] Hammond KB, Turcios NL, Gibson LE (1994). Clinical evaluation of the macroduct sweat collection system and conductivity analyzer in the diagnosis of cystic fibrosis. *Journal of Pediatrics*.

[23] Walling HW, Swick BL (2011). Treatment options for hyperhidrosis. *American Journal of Clinical Dermatology*.

[24] Kinoshita T, Shibaji T, Umino M (2003). Transdermal delivery of lidocaine in vitro by alternating current. *Journal of Medical and Dental Sciences*.

[25] Haga H, Shibaji T, Umino M (2005). Lidocaine transport through living rat skin using alternating current. *Medical and Biological Engineering and Computing*.

[26] Izumikawa H (2005). Lidocaine transportation through a cellophane membrane by wide range AC frequencies. *Kokubyo Gakkai Zasshi*.

[27] Ogami S, Hayashi S, Shibaji T, Umino M (2008). Pentazocine transport by square-wave AC iontophoresis with an adjusted duty cycle. *Journal of Medical and Dental Sciences*.

[28] Hayashi S, Ogami S, Shibaji T, Umino M (2009). Lidocaine transport through a cellophane membrane by alternating current iontophoresis with a duty cycle. *Bioelectrochemistry*.

